# GO/Cysteamine-Modified Screen-Printed Carbon Electrode for Square-Wave Anodic Stripping Voltammetric Determination of Cadmium in Fortified Mango and Avocado Digestates

**DOI:** 10.3390/bios16100544

**Published:** 2026-09-29

**Authors:** Miguel Angel Lozada Rufino, Eulogia Isabel Zapata Peña, Gonzalo Manuel Delgado Valdiviezo, Rosa Gisela Ortiz Castillo, Grecia Xiomara Herrera Gavilan, David César Ardiles Saravia, Luis Alfredo Espinoza-Espinoza, Karina Silvana Gutiérrez-Valverde

**Affiliations:** 1 Functional Foods and Bioengineering Research Group, National University of Frontera, Av. San Hilarión No. 101, Sullana 20101, Peru; lozada.miguel@ufabc.edu.br (M.A.L.R.); 2020103035@unf.edu.pe (E.I.Z.P.); 2018103026@unf.edu.pe (G.M.D.V.); rortizc@unf.edu.pe (R.G.O.C.); 2014103026@unf.edu.pe (G.X.H.G.); 2 Center of Natural and Human Sciences, Federal University of ABC, Santo André 09210-580, SP, Brazil; 3 Faculty of Engineering of Food Industries and Biotechnology, Academic Department of Food Industries Engineering, National University of Frontera, Av. San Hilarión No. 101, Sullana 20101, Peru; 4 3R Aerospace & Nanotechnology, 38000 Grenoble, France; davidcesar@yahoo.com; 5 Department of Engineering, National University of Barranca, Barranca 15169, Peru; lespinozae@unab.edu.pe

**Keywords:** Cadmium, electrochemical sensor, reduced graphene oxide, cysteamine, SWASV, mango, avocado

## Abstract

Cadmium contamination in fruits is a relevant food-safety concern because of its toxicity, environmental persistence, and potential transfer from agricultural soils to edible plant tissues. This study developed a screen-printed carbon electrode modified with graphene oxide and cysteamine (SPCE/GO–cysteamine) to determine Cd(II) by square-wave anodic stripping voltammetry (SWASV). Two surface-modification routes, GO-cysteamine and GO-rGO-cysteamine, and GO concentrations of 0.1, 0.2, and 0.5 mg mL^−1^ were compared by cyclic voltammetry. The direct GO-cysteamine route at 0.2 mg mL^−1^ produced the highest CV response among the tested conditions and was selected for Cd(II) determination. Raman spectroscopy FTIR spectroscopy and scanning electron microscopy were used to characterize the graphene-based materials employed during surface optimization. Cd(II) concentrations from 1 to 10 µg L^−1^ were experimentally evaluated; with a calculated LOQ of 0.74 µg L^−1^, the entire experimentally evaluated range of 1–10 µg L^−1^, with a sensitivity of 11.30 µA (µg L^−1^)^−1^, R^2^ = 0.9892, a limit of detection of 0.25 µg L^−1^ and a limit of quantification of 0.74 µg L^−1^. Fortified Kent mango and Hass avocado digestates also showed concentration-dependent responses, with R^2^ values of 0.9799 and 0.9788, respectively, and relative standard deviations below 5%. These results demonstrate the feasibility of the SPCE/GO–cysteamine platform for Cd(II) determination in digested fruit matrices and support its further development as a portable screening approach for food-quality control.

## 1. Introduction

Cadmium (Cd) contamination in the food chain represents a significant food-safety concern because of its toxicity, environmental persistence, and capacity to accumulate in agricultural soils and subsequently enter edible plant tissues [1,2]. Chronic exposure to Cd is primarily associated with adverse effects on renal function and bone metabolism, making dietary exposure an important issue for public-health surveillance [2,3,4,5,6]. Fruits intended for domestic consumption and international trade therefore require appropriate monitoring strategies, particularly in production areas where soil composition and agricultural practices may affect Cd availability and plant uptake [3,4,5].

The European Union regulates maximum Cd concentrations in food through Commission Regulation (EU) 2023/915 [7]. The maximum level established for mango is 0.020 mg kg^−1^ fresh weight, whereas fruits not specifically included in that category are subject to a maximum level of 0.050 mg kg^−1^ fresh weight [7]. These regulatory requirements, together with the spatial variability reported for Cd in agricultural soils, reinforce the need for analytical strategies capable of supporting routine monitoring throughout fruit-production and commercialization chains [5,8].

Conventional techniques such as inductively coupled plasma mass spectrometry (ICP-MS), inductively coupled plasma optical emission spectrometry (ICP-OES), and atomic absorption spectroscopy (AAS) provide high sensitivity and analytical reliability for trace-metal determination [9,10]. Nevertheless, their routine implementation generally requires centralized laboratory infrastructure, specialized personnel, sample mineralization, and relatively expensive instrumentation [1,9,10,11,12]. Electrochemical methods have therefore attracted considerable interest as complementary screening approaches because they can combine relatively low instrumentation costs, reduced reagent consumption, portability, and compatibility with disposable electrodes [9,11,12,13].

Among electroanalytical approaches, anodic stripping voltammetry is particularly suitable for trace-metal determination because it combines an electrochemical preconcentration step with subsequent anodic stripping of the accumulated metal. Square-wave anodic stripping voltammetry (SWASV) further provides rapid potential scanning and high sensitivity while remaining compatible with compact potentiostats and screen-printed electrodes [12,13,14,15]. Screen-printed carbon electrodes (SPCEs) are especially attractive because their working surfaces can be readily modified with nanostructured carbon materials and chemical recognition groups to tailor analyte accumulation and interfacial electron-transfer processes [9,13,16].

Graphene oxide (GO) offers a high surface area and contains oxygen-bearing functionalities, including carboxyl groups, that can be exploited for further chemical modification [17,18]. In the present approach, carbodiimide chemistry was used to promote coupling between surface carboxyl groups and the amino functionality of cysteamine. Cysteamine is a bifunctional molecule containing amino (–NH_2_) and thiol (–SH) groups, and surface-exposed sulfur-containing groups can interact strongly with Cd(II) [19,20,21]. Previous studies have demonstrated that cysteamine-containing carbon interfaces can enhance the electrochemical response toward Cd(II) and can be incorporated into disposable electrochemical platforms for trace-metal analysis [19,20]. The interaction between sulfur donors and Cd(II) is also consistent with hard–soft acid–base considerations, since Cd(II) behaves as a relatively soft Lewis acid with affinity toward sulfur-containing ligands [21,22,23].

The analytical behavior of such platforms in food matrices, however, cannot be inferred solely from measurements in aqueous standards. Mango (*Mangifera indica* L.) contains sugars, organic acids, fibers, and phenolic compounds, whereas avocado (*Persea americana*) contains a substantial lipid fraction in addition to other organic constituents [24,25,26]. Even after sample digestion, differences in residual matrix composition and solution chemistry may modify background current, metal availability, interfacial accumulation, and stripping response. Recent studies have also demonstrated spatial variability of Cd in avocado-producing soils in Peru, emphasizing the relevance of analytical monitoring at the crop and fruit levels [5,27].

Therefore, this study aimed to develop and evaluate a DropSens C-110 screen-printed carbon electrode modified with GO and functionalized with cysteamine for Cd(II) determination by SWASV. Two surface-fabrication routes, GO-cysteamine and GO-rGO-cysteamine, were initially compared, together with GO concentrations of 0.1, 0.2, and 0.5 mg mL^−1^. The selected platform was subsequently evaluated using Cd(II) standard solutions and fortified digestates of Kent mango and Hass avocado. The study specifically examined electrochemical response, linearity, sensitivity, detection and quantification limits, and repeatability to assess the feasibility of the platform as a portable electrochemical approach for Cd(II) screening in digested fruit simples.

## 2. Materials and Methods

### 2.1. Chemicals and Reagents

Cysteamine (≥98%, Sigma-Aldrich, St. Louis, MO, USA) was used as the surface-functionalizing molecule. *N*-Hydroxysuccinimide (NHS), 1-ethyl-3-(3-dimethylaminopropyl) carbodiimide (EDC), 2-(N-morpholino)ethanesulfonic acid (MES) buffer, phosphate-buffered saline (PBS), potassium chloride (KCl), and a potassium hexacyanoferrate redox probe were used during electrode activation, functionalization, and electrochemical characterization.

Nitric acid (HNO_3_, 65%, Suprapur, Merck, Darmstadt, Germany) and hydrogen peroxide (H_2_O_2_, 30%, Merck) were used for fruit-sample digestion. Hydrochloric acid (HCl, 0.05 M) was used for preparation of the electrochemical measurement medium. Ultrapure water with a resistivity of 18.2 MΩ cm was used throughout solution preparation, washing, and dilution procedures.

Cd(II) working standards were prepared by serial dilution of a certified 1000 mg L^−1^ Cd stock solution. Glassware used for trace-metal preparation and analysis was previously treated with 10% HNO_3_ for 24 h, rinsed three times with ultrapure water, and dried at 60 °C before use.

Screen-printed carbon electrodes (C-110, DropSens, Oviedo, Spain) were used as the electrochemical platform. The electrode consisted of a carbon working electrode, a carbon counter electrode, and an integrated silver pseudo-reference electrode. An aqueous graphene oxide (GO) suspension supplied at 2 mg mL^−1^ was used for modification of the working electrode.

### 2.2. Electrochemical Instrumentation

Electrochemical measurements were performed using a portable Metrohm DropSens potentiostat (Metrohm DropSens, Oviedo, Spain) controlled through DropView 8400 software. Cyclic voltammetry (CV) was employed for initial electrode characterization and comparison of the different surface-modification conditions. Square-wave anodic stripping voltammetry (SWASV) was subsequently used for Cd(II) determination.

The C-110 electrodes were operated using their integrated three-electrode configuration consisting of the modified carbon working electrode, carbon counter electrode, and silver pseudo-reference electrode.

### 2.3. Pretreatment and Modification of the C-110 Working Electrode

Before modification, the C-110 electrodes were rinsed with ultrapure water and ethanol and subsequently dried. Electrochemical conditioning was performed in 0.1 M KCl by cyclic voltammetric scanning to stabilize the electrochemical response. The initial electron-transfer behavior of each electrode was evaluated using a 5 mM hexacyanoferrate redox probe in 0.1 M KCl.

GO working dispersions at concentrations of 0.1, 0.2, and 0.5 mg mL^−1^ were prepared from the commercial 2 mg mL^−1^ suspension. An aliquot of each dispersion was deposited onto the carbon working-electrode area by drop-casting and allowed to dry at room temperature before subsequent surface activation.

The three GO concentrations were evaluated to determine the surface loading that provided the most suitable voltammetric behavior following cysteamine functionalization.

### 2.4. Surface Activation and Cysteamine Functionalization

Cysteamine immobilization was performed using EDC/NHS-mediated activation of oxygen-containing surface groups on GO. NHS and EDC solutions were prepared separately in 50 mM MES buffer at pH 6.0. NHS (3.4 mg) was dissolved in 200 µL of MES buffer to obtain approximately 150 mM, whereas EDC (3.8 mg) was dissolved in 200 µL of the same buffer to obtain approximately 100 mM. Equal volumes of the EDC and NHS solutions were mixed immediately before use. An 8 µL aliquot of the activation mixture was deposited onto the GO-modified working electrode and incubated for 45 min at room temperature. This activation step was used to promote formation of reactive intermediates from surface carboxyl groups for subsequent coupling with the amino group of cysteamine.

After activation, the electrode was washed with 10 mM PBS (pH 7.4) to remove excess EDC/NHS reagents and dried under a nitrogen stream. A 50 mM cysteamine solution was subsequently prepared in 10 mM PBS (pH 7.4). An 8 µL aliquot was deposited onto the activated working electrode and incubated for 120 min at room temperature. Following functionalization, excess cysteamine was removed by successive washing with PBS and ultrapure water, and the electrode was dried under nitrogen.

Residual activated sites were blocked by applying 8 µL of 150 mM ethanolamine for 60 min. The electrodes were subsequently rinsed with ultrapure water and dried before electrochemical characterization.

### 2.5. Optimization of the Electrode-Modification Route

Two surface-modification routes were investigated. In the first route, GO deposited on the working electrode was directly activated with EDC/NHS and subsequently functionalized with cysteamine (GO-cysteamine). In the second route, deposited GO was electrochemically reduced to rGO by chronoamperometry at −1.8 V for 60 s before the cysteamine-functionalization procedure (GO-rGO-cysteamine).

The electrochemical behavior of the two routes was compared by cyclic voltammetry. The comparison was intended to determine whether electrochemical reduction in GO before functionalization improved or impaired the interfacial electrochemical response.

After selection of the surface-modification route, the influence of GO concentration was examined using 0.1, 0.2, and 0.5 mg mL^−1^ dispersions. For each condition, the response of the unmodified C-110 electrode, the GO-modified electrode, and the GO/cysteamine-functionalized electrode was compared by CV. Based on the resulting electrochemical response, 0.2 mg mL^−1^ GO was selected for subsequent Cd(II) measurements.

Although electrochemical reduction can improve conductivity, it also decreases oxygen-containing groups available for EDC/NHS-mediated functionalization; together with the more pronounced cathodic response observed by CV, this supported selection of the non-reducing GO–cysteamine route.

### 2.6. Square-Wave Anodic Stripping Voltammetry for Cd(II)

Cd(II) determination was performed by SWASV using the selected C-110/GO–cysteamine electrode. Standard Cd(II) solutions covering the range from 1 to 10 µg L^−1^ were analyzed.

The SWASV procedure consisted of a cathodic deposition step followed by anodic stripping. Cd(II) was electrodeposited at −1.10 V for 300 s. After deposition, an equilibration time of 5 s was applied before the stripping scan. The square-wave frequency was 25 Hz, the pulse amplitude was 0.030 V, and the potential step was 0.005 V. The stripping scan was performed from −1.10 to 0.00 V.

The analytical signal was defined as the Cd-associated stripping peak current obtained from the SWASV response. Calibration curves were constructed by plotting peak current against Cd(II) concentration.

### 2.7. Raman Spectroscopy, FTIR Spectroscopy and Scanning Electron Microscopy

Graphene-based materials generated during the electrode-modification study were characterized by Raman spectroscopy, FTIR-ATR spectroscopy, and scanning electron microscopy (SEM).

Raman measurements were performed using a Horiba Jobin Yvon T64000 Raman spectrometer (Villeneuve d’Ascq, France) at 532 nm. Spectra were collected over the region containing the characteristic D and G bands of graphene-derived materials. The D and G band responses were used to calculate the (I_D_/I_G_) intensity ratio as a comparative indicator of structural disorder and changes in the sp^2^ carbon domains.

FTIR-ATR spectra were recorded using a PerkinElmer spectrometer (Shelton, CT, USA) equipped with a PIKE GladiATR accessory. Measurements were acquired in transmittance mode over the 4000–450 cm^−1^ range, using 64 accumulations and a spectral resolution of 4 cm^−1^. The spectra were used to compare the surface-chemical features of the GO-based samples before and after cysteamine functionalization.

Surface morphology was evaluated using a JEOL JSM-6610LA scanning electron microscope (Tokyo, Japan) operated at an accelerating voltage of approximately 10–15 kV. The graphene-based surfaces were examined at different magnifications to evaluate sheet morphology, folding, aggregation, surface heterogeneity, and changes associated with the modification procedure. When required for SEM imaging, the samples were coated with a thin conductive layer to minimize charging.

### 2.8. Fruit Samples and Sample Preparation

Kent mango (*Mangifera indica* L., and Kent) and Hass avocado (*Persea americana*, cv. Hass) were obtained from Calle & Palacios SAC in the Piura region of Peru. Fruits were selected at commercial maturity and only specimens with intact external appearance and no visible deterioration were included.

Three lots were considered for each fruit, with 10 fruits per lot. Thus, 30 mangoes and 30 avocados were included, corresponding to 60 individual fruits. Pulp from the fruits was processed to obtain composite samples representative of each cultivar and lot.

The pulp samples were dried at 60 °C until constant mass, pulverized, and homogenized before digestion. A 0.500 g portion of dried sample was transferred to the digestion vessel and mixed with 5.0 mL of concentrated HNO_3_ (65%) and 2.0 mL of H_2_O_2_ (30%). The mixture was allowed to predigest for 15 min at room temperature before thermal treatment.

Samples were subsequently subjected to the thermal digestion procedure at 200 °C for 20 min. After cooling to room temperature, each digest was quantitatively transferred to a 25.0 mL volumetric flask and diluted to volume with ultrapure water. Aliquots intended for electrochemical analysis were subsequently diluted or adjusted using 0.05 M HCl before SWASV measurement.

Because the mass used for digestion corresponded to dried pulp, concentrations converted from digestate units to sample units in this study refer to the dry sample mass used during digestion. Conversion to a fresh-weight basis requires the experimentally determined moisture content of each fruit matrix.

### 2.9. Evaluation of Analytical Performance

The analytical performance of the SPCE/GO–cysteamine platform was evaluated in terms of linearity, sensitivity, limit of detection (LOD), limit of quantification (LOQ), and repeatability. Calibration curves were constructed by plotting the Cd(II) stripping peak current (I_p_) against the corresponding Cd(II) concentration (C). The relationship between analytical signals and concentration was described by linear regression according to:

$$I_{p}=mC+b$$

where (m) represents the slope of the calibration curve and was used as the analytical sensitivity, while (b) corresponds to the regression intercept.

The LOD and LOQ were estimated from the standard deviation of the blank response (σ) and the slope of the corresponding calibration curve (m) according to:

$$LOD=\frac{3.3\sigma }{m}$$

$$LOQ=\frac{10\sigma }{m}$$

where “σ” is the standard deviation of the blank response and “m” is the calibration slope. For the standard calibration, σblank was obtained from five replicate measurements of the 0.05 M HCl reagent blank. The reported LOD and LOQ therefore represent reagent-blank-based estimates rather than full-method detection and quantification limits.

For calibration in standard solutions, Cd(II) concentrations ranging from 1 to 10 µg L^−1^ were evaluated. Fortified Kent mango digestates were analyzed at added Cd(II) concentrations of 1, 2, and 3 µg L^−1^, whereas fortified Hass avocado digestates were evaluated at 1, 2, 3, and 4 µg L^−1^. Each fortified concentration and the corresponding reagent blank were measured in five replicates (*n* = 5). Repeatability was expressed as the relative standard deviation (RSD, %) of replicate measurements.

For fruit digestates, analytical limits expressed in µg L^−1^ were converted to concentrations referred to the digested sample mass by considering a sample mass of 0.500 g and a final digest volume of 25.0 mL. The conversion was performed according to:

$${C_{sample}\left(mg{kg}^{-1}\right)=C}_{difest}\left(\mu gL^{-1}\right)\times \frac{0.025\;L}{0.0005\;kg}\times \;10^{-3}\times \frac{mg}{\mu g}$$

which results in:

$$C_{sample}\left(mg{kg}^{-1}\right)=C_{difest}\left(\mu g,L^{-1}\right)\;\times \;0.05$$

Therefore, a conversion factor of 0.05 was applied to convert Cd concentrations measured in the final digest from µg L^−1^ to mg kg^−1^ referred to the dry sample mass used for digestion.

The measurements performed using fortified fruit digestates were used to evaluate the electrochemical response and repeatability of the sensor in the presence of the mango and avocado matrices. Because Cd(II) fortification was performed after the digestion step, these experiments evaluate the performance of the electrochemical determination in the digested matrix and should not be interpreted as recovery of the complete sample-preparation and digestion procedure.

## 3. Results

### 3.1. Optimization of Electrode Modification

#### 3.1.1. Comparison of the GO-Cysteamine and GO-rGO-Cysteamine Routes

**Figure 1:**
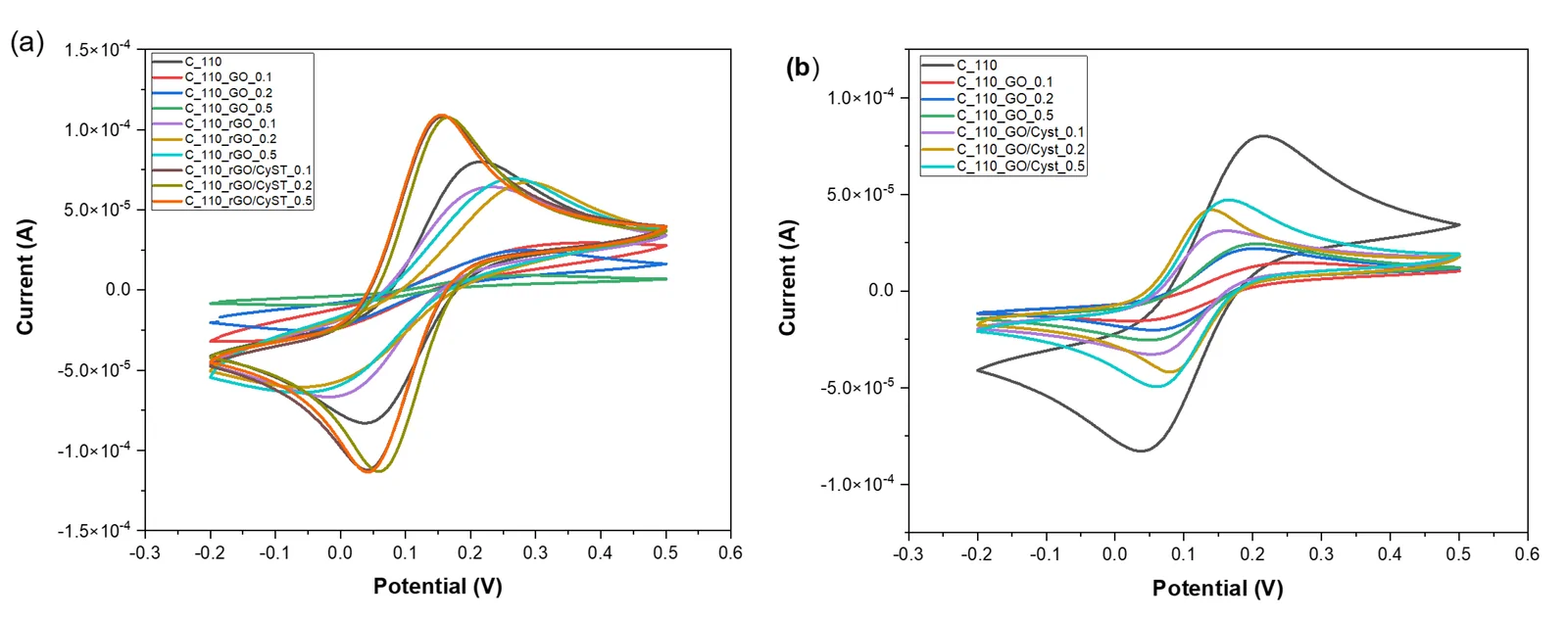
Cyclic voltammograms obtained in 5 mM hexacyanoferrate/0.1 M KCl comparing (**a**) the GO-rGO-cysteamine and (**b**) GO-cysteamine modification routes on the C-110 screen-printed carbon electrode. GO concentrations were 0.1, 0.2, and 0.5 mg mL^−1^. Potentials are reported versus the integrated Ag pseudo-reference electrode. Scan rate: 80 mV s^−1^.

Two modification routes were initially evaluated by cyclic voltammetry: direct functionalization of GO with cysteamine (GO-cysteamine) and electrochemical reduction in GO before cysteamine functionalization (GO-rGO-cysteamine). The two configurations produced distinguishable voltammetric profiles (Figure 1).

The GO-rGO-cysteamine configuration showed anodic peak currents of approximately +80 to +90 µA and cathodic peak currents ranging from approximately −30 to −50 µA (Table S1). The corresponding anodic peak potentials were observed between +0.20 and +0.30 V, while the cathodic response occurred at approximately −0.05 V.

In comparison, the GO-cysteamine configuration maintained anodic peak currents of approximately +80 to +90 µA but produced higher cathodic current magnitudes, ranging from approximately −70 to −90 µA. The anodic peak occurred between approximately +0.05 and +0.20 V, while the cathodic response was located between −0.05 and −0.10 V.

Thus, the most pronounced difference between the two modification routes was observed in the cathodic response, which was approximately 1.5–2 times greater in magnitude for the GO-cysteamine configuration. Based on this preliminary CV comparison, the direct GO–cysteamine route was selected for subsequent optimization and Cd(II) evaluation.

#### 3.1.2. Effect of GO Concentration

**Figure 2:**
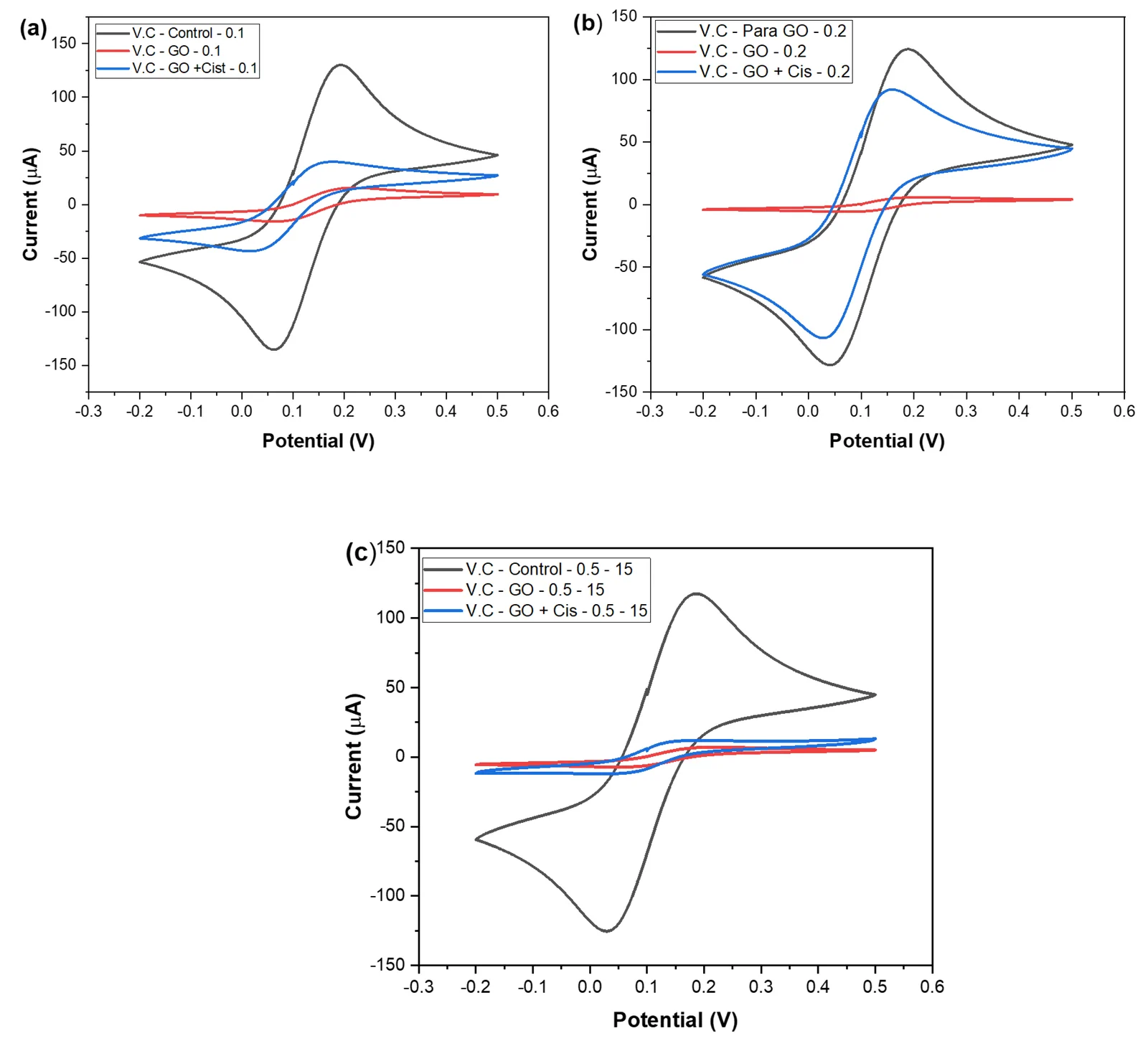
Cyclic voltammograms obtained in 5 mM hexacyanoferrate/0.1 M KCl during optimization of GO concentration at (**a**) 0.1, (**b**) 0.2, and (**c**) 0.5 mg mL^−1^. Each panel compares the unmodified C-110, GO-modified, and GO/cysteamine-functionalized electrodes. Potentials are reported versus the integrated Ag pseudo-reference electrode. Scan rate: 80 mV s^−1^.

After selecting the GO-cysteamine modification route, GO concentrations of 0.1, 0.2, and 0.5 mg mL^−1^ were evaluated by cyclic voltammetry (Figure 2). For each concentration, the response of the unmodified C-110 electrode was compared with those obtained after GO deposition and after cysteamine functionalization.

At 0.1 mg mL^−1^ GO, the unmodified electrode exhibited anodic and cathodic currents of approximately +125 and −135 µA, respectively. Deposition of GO markedly decreased the voltammetric response to current magnitudes below approximately ±30 µA. After cysteamine functionalization, the response partially recovered, reaching approximately +50 µA in the anodic direction and −75 µA in the cathodic direction.

At 0.2 mg mL^−1^ GO, cysteamine functionalization produced the highest current response among the three GO concentrations investigated. The anodic and cathodic currents reached approximately +75 and −100 µA, respectively, with the cathodic peak located close to −0.10 V.

Increasing the GO concentration to 0.5 mg mL^−1^ resulted in lower current responses than those obtained at 0.2 mg mL^−1^. Therefore, based on the comparative cyclic voltametric response, 0.2 mg mL^−1^ GO was selected as the working concentration for subsequent preparation of the SPCE/GO–cysteamine platform.

### 3.2. Structural and Morphological Characterization

#### 3.2.1. Raman Spectroscopy

**Figure 3:**
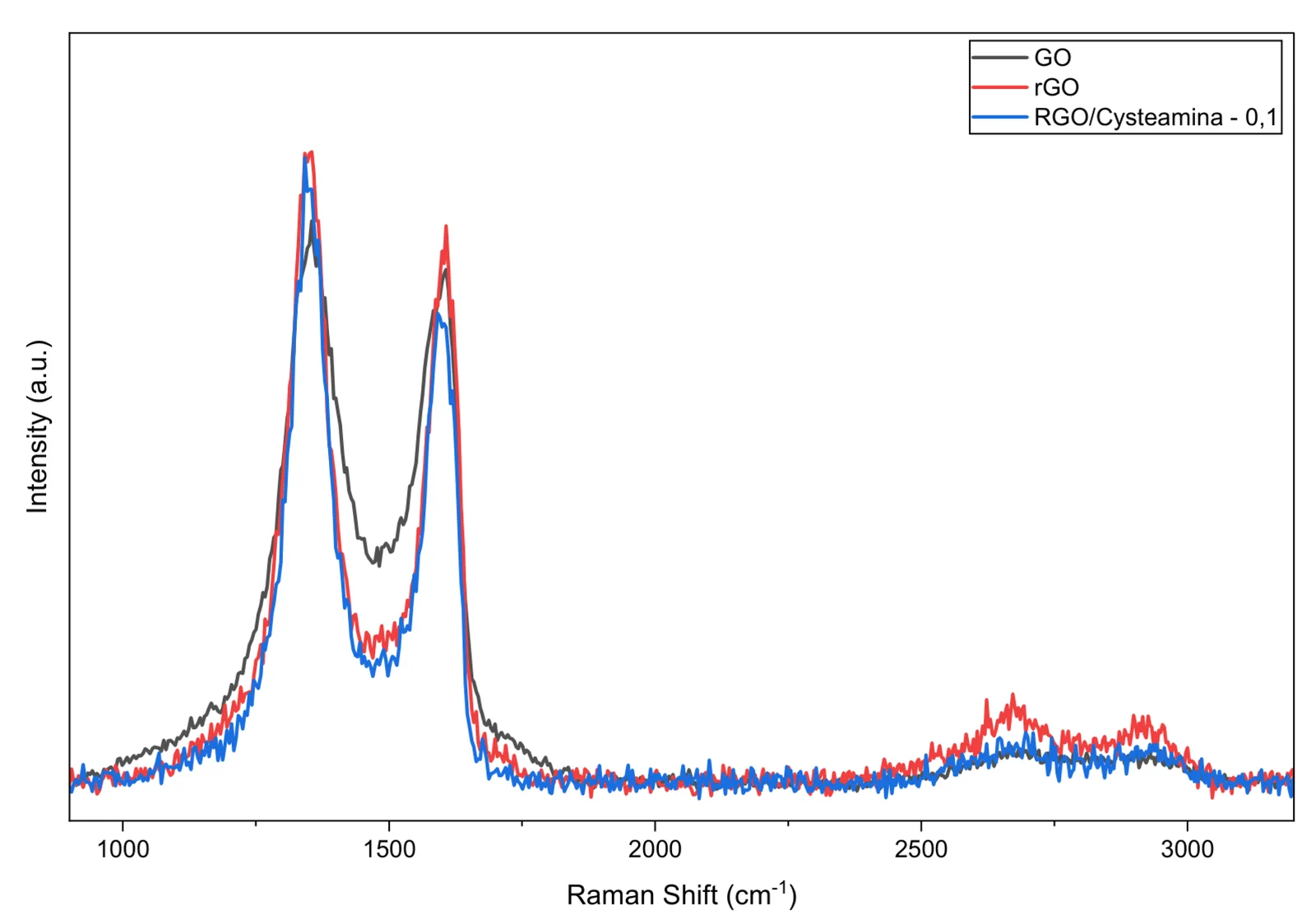
Raman spectra of GO, rGO, and rGO/cysteamine showing the characteristic D and G bands.

The Raman spectra of GO, rGO, and rGO/cysteamine exhibited the characteristic D and G bands of graphene-derived carbon materials (Figure 3). The D band was observed at approximately 1350 cm^−1^, whereas the G band was located within the 1580–1600 cm^−1^ region.

The estimated D- and G-band intensities were similar for rGO and rGO/cysteamine, respectively. Accordingly, the I_D_/I_G_ ratio increased from 0.94 for GO to 1.08 for rGO and 1.15 for rGO/cysteamine.

These results demonstrate progressive changes in the relative contribution of the D and G bands following the reduction and functionalization steps.

#### 3.2.2. FTIR Spectroscopy

FTIR spectra of the bare SPCE C-110, SPCE/GO, and SPCE/GO–cysteamine surfaces showed distinct changes after GO deposition and subsequent cysteamine treatment (Figure S1). Differences were observed mainly in the C–O/C–O–C region (~1000–1030 cm^−1^), the C–N/C–O region (~1200–1250 cm^−1^), the C=C/carbonyl-related region (~1600–1630 cm^−1^), and the broad O–H/N–H region (~3200–3350 cm^−1^). The spectral changes observed between SPCE/GO and SPCE/GO–cysteamine support modification of the GO-coated surface following cysteamine treatment.

#### 3.2.3. Scanning Electron Microscopy

**Figure 4:**
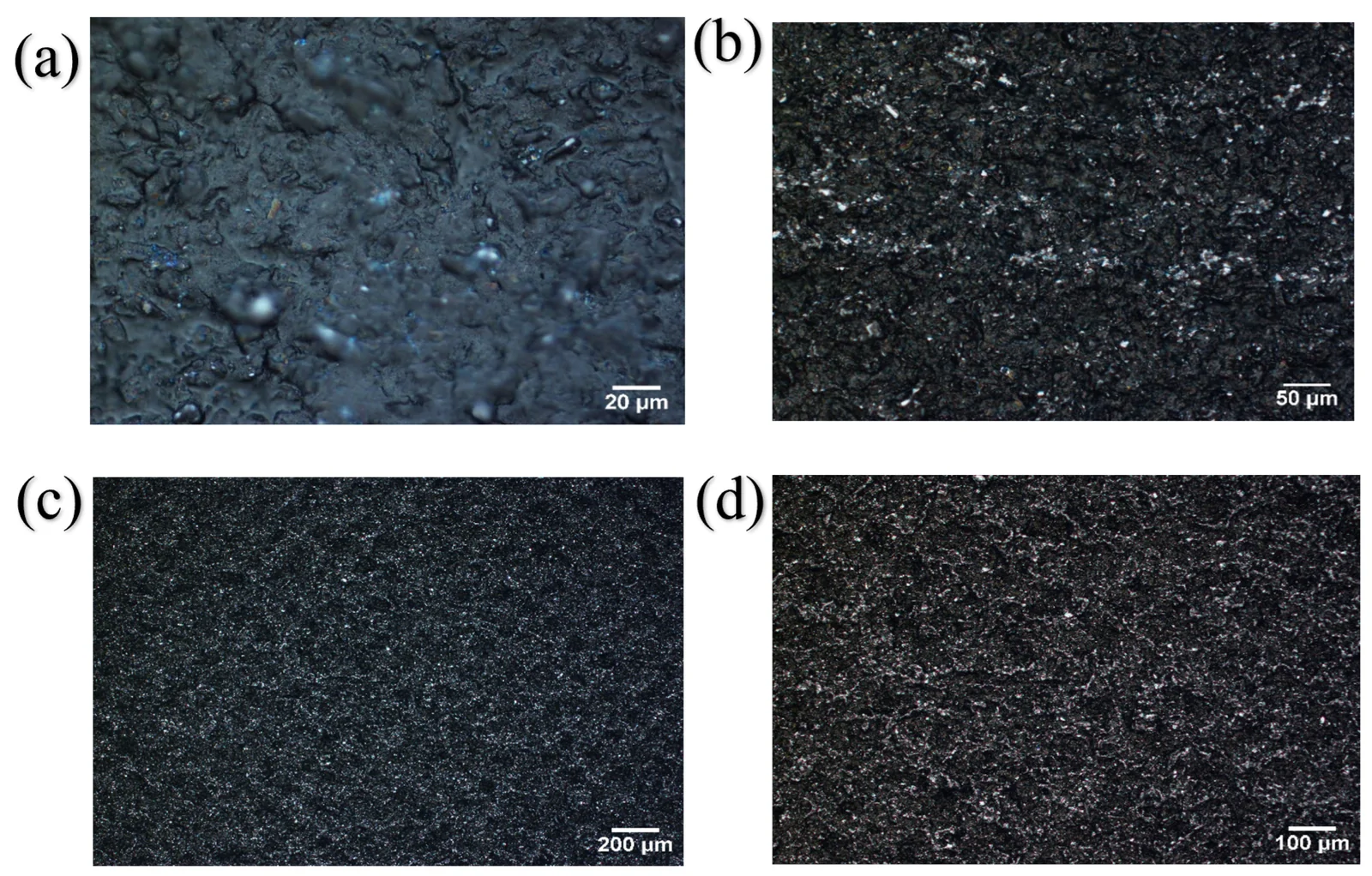
SEM micrographs of graphene oxide obtained at different magnifications, showing the lamellar, folded, and heterogeneous surface morphology. (**a**) 200 µm, (**b**) 100 µm, (**c**) 50 µm and (**d**) 20 µm.

SEM micrographs of GO showed a predominantly lamellar morphology consisting of overlapping sheets with irregular edges and pronounced folding. At lower magnification, the deposited material appeared relatively continuously across the analyzed region, whereas higher-magnification images revealed wrinkles, folds, and heterogeneous surface features characteristic of the layered GO structure (Figure 4).

The surface therefore exhibited a rough and non-uniform morphology with multiple overlapping graphene-derived sheets.

### 3.3. Electrochemical Response Toward Cd(II) in Standard Solutions

**Figure 5:**
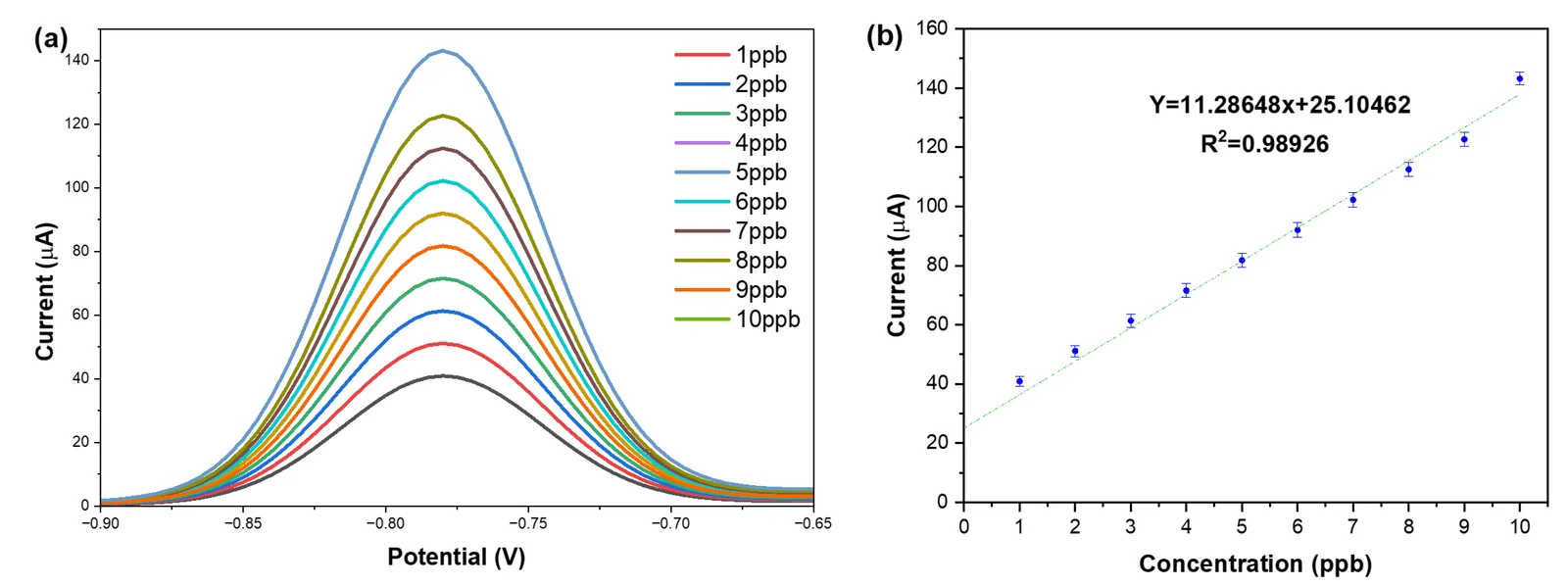
(**a**) SWASV responses of the SPCE/GO–cysteamine platform for Cd(II) concentrations from 1 to 10 µg L^−1^ and (**b**) corresponding calibration curve.

The analytical response of the SPCE/GO–cysteamine platform toward Cd(II) was evaluated by SWASV over the concentration range from 1 to 10 µg L^−1^ (Figure 5a). The stripping response increased progressively with increasing Cd(II) concentration. Linear regression of the peak current against Cd(II) concentration yielded:

I_p_ = 11.30 C + 25.1046

where I_p_ is the peak current in µA and C is the Cd(II) concentration in µg L^−1^. The calibration curve showed a coefficient of determination of R^2^ = 0.9893 (Figure 5b). The corresponding analytical sensitivity, defined as the slope of the calibration curve, was 11.30 µA (µg L^−1^)^−1^. Using the standard deviation of the blank and the calibration slope (Table S2), the LOD and LOQ were estimated as 0.25 and 0.74 µg L^−1^, respectively. Accordingly, the experimentally evaluated calibration interval of 1–10 µg L^−1^ was entirely above the calculated LOQ.

### 3.4. Cd(II) Determination in Mango and Avocado Matrices

**Figure 6:**
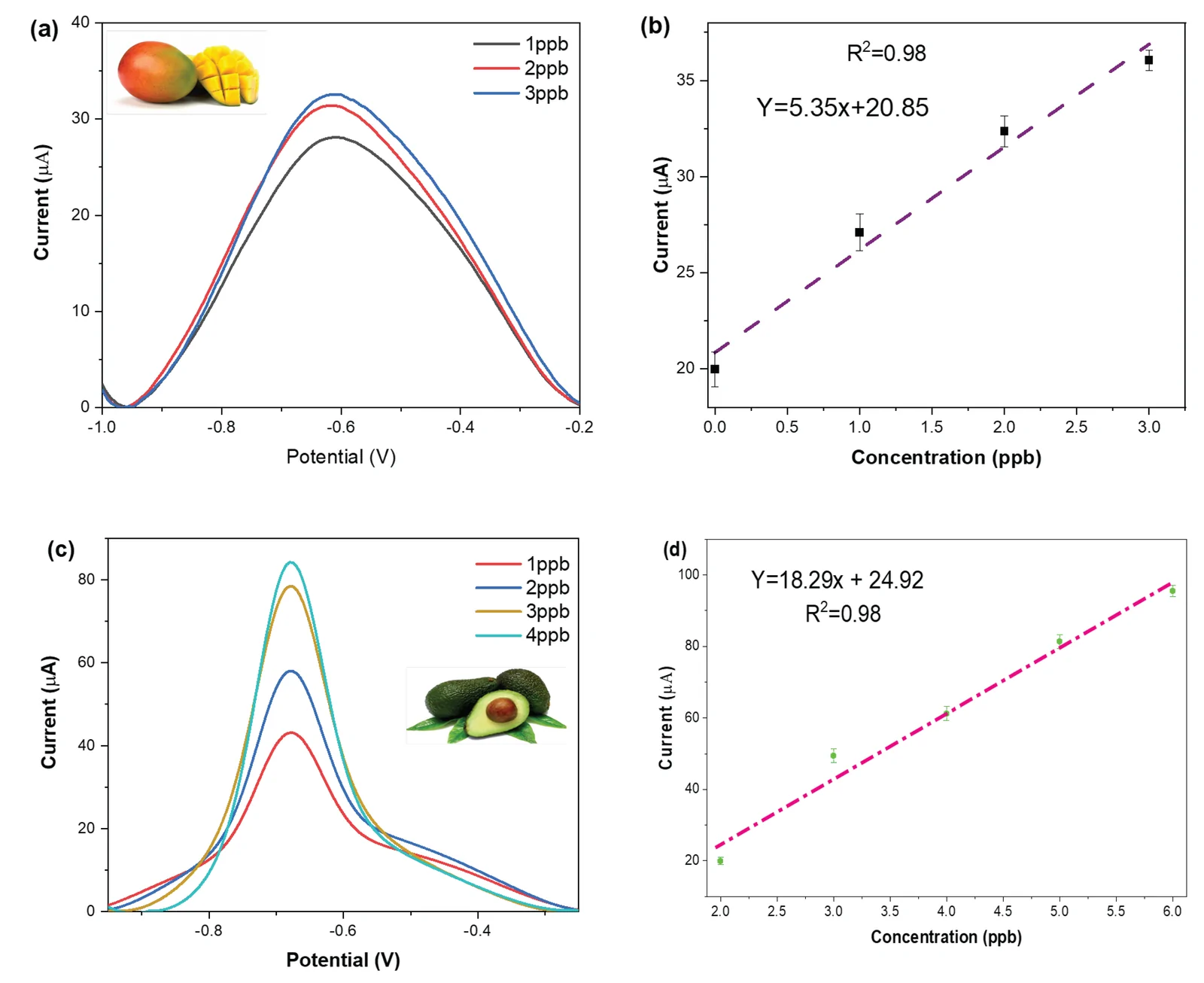
(**a**) SWASV response and calibration curve obtained for Cd(II)-fortified Kent mango digestates. (**b**) calibration curve obtained for Cd(II)-fortified Kent mango digestates (**c**) SWASV response and calibration curve obtained for Cd(II)-fortified Hass avocado digestates. (**d**) calibration curve obtained for Cd(II)-fortified Hass avocado digestates.

The electrochemical response of the SPCE/GO–cysteamine platform was subsequently evaluated in fortified Kent mango digestates. Added Cd(II) concentrations of 1, 2, and 3 µg L^−1^ produced mean peak currents of 27.10, 32.35, and 36.05 µA, respectively (Figure 6a). The corresponding calibration relationship was:

I_p_ = 5.35 C + 20.8491

with R^2^ = 0.9799 (Figure 6). The sensitivity obtained in the mango digestate was therefore 5.35 µA (µg L^−1^)^−1^. The RSD decreased from 3.50% at 1 µg L^−1^ to 2.51% at 2 µg L^−1^ and 1.50% at 3 µg L^−1^. All RSD values obtained for the fortified mango digestate were below 5% (Table S3).

The estimated LOD and LOQ in the final digest were 0.56 and 1.68 µg L^−1^, respectively. Considering the digestion of 0.50 g of dry sample to a final volume of 25.0 mL, these values correspond to 0.0278 and 0.0841 mg kg^−1^, respectively, referred to the dry sample mass used for digestion. The 1 µg L^−1^ fortification level was below the calculated LOQ of 1.68 µg L^−1^ and should therefore be interpreted as a detectable response rather than a reliably quantifiable concentration.

Hass avocado digestates were evaluated at added Cd(II) concentrations of 1, 2, 3, and 4 µg L^−1^. The corresponding mean peak currents were 49.45, 61.25, 81.35, and 95.50 µA, respectively (Figure 5c). Linear regression yielded:

I_p_ = 18.30 C + 24.9163

with R^2^ = 0.9788 (Figure 5d). The corresponding sensitivity was 18.30 µA (µg L^−1^)^−1^. The RSD values were 4.00%, 3.19%, 2.40%, and 1.60% for 1, 2, 3, and 4 µg L^−1^ Cd(II), respectively. As observed for mango, all replicate measurements showed RSD values below 5% (Table S4).

The LOD and LOQ obtained for the avocado digestate were 0.17 and 0.52 µg L^−1^, respectively. After conversion according to the sample mass and final digest volume, these values corresponded to 0.00866 and 0.0262 mg kg^−1^, respectively, on the dry-sample basis used in the digestion procedure.

### 3.5. Comparison of the Analytical Response Between Matrices

The calibration behavior of the SPCE/GO–cysteamine platform differed among the three analytical media. The slope obtained in standard solutions was 11.30 µA (µg L^−1^)^−1^, compared with 5.35 µA (µg L^−1^)^−1^ in Kent mango digestate and 18.30 µA (µg L^−1^)^−1^ in Hass avocado digestate. Thus, the sensitivity in mango digestate was approximately 53% lower than that obtained with standard solutions. In contrast, the slope obtained in avocado digestate was approximately 62% higher than that obtained in standard solutions and approximately 3.4 times higher than that measured in mango digestate.

Despite these differences in calibration slope, concentration-dependent responses were obtained in both fruit digestates over the investigated ranges, with RSD values below 5%. The main analytical parameters obtained for the standard solutions and fortified fruit digestates are summarized in Table 1.

## 4. Discussion

The electrochemical optimization showed that the direct GO-cysteamine route produced a more pronounced cathodic response than the GO-rGO-cysteamine route. While both configurations exhibited anodic currents of approximately +80 to +90 µA, the cathodic current increased in magnitude from approximately −30 to −50 µA for GO-rGO-cysteamine to −70 to −90 µA for GO-cysteamine. This behavior suggests that retaining the oxygen-containing functionalities of GO before cysteamine immobilization is advantageous for the interfacial modification employed in this study. GO contains carboxyl and other oxygenated groups that provide chemically accessible sites for subsequent functionalization [17,18,22]. Under EDC/NHS activation, surface carboxyl groups can form reactive intermediates capable of coupling with the amino group of cysteamine, whereas electrochemical reduction in GO decreases the overall abundance of oxygen-containing functionalities [18,22]. Therefore, the lower response obtained after GO reduction may be associated with a lower availability of coupling sites. Nevertheless, because cysteamine surface coverage and free thiol density were not directly quantified, this mechanism should be regarded as an interpretation consistent with the observed electrochemical behavior rather than direct evidence of molecular surface coverage.

The proposed role of cysteamine in Cd(II) accumulation is associated with the affinity of sulfur-containing groups for Cd(II). Previous studies have shown that cysteamine-functionalized carbon interfaces can enhance the electrochemical response toward Cd(II), which has been attributed to interactions between accessible sulfur-containing groups and the metal ion [19,20]. In the proposed GO–cysteamine architecture, the amino functionality of cysteamine participates in its attachment to activated oxygen-containing groups on GO, while the sulfur-containing end remains available at the electrode–solution interface. During the cathodic deposition step of SWASV, these sites may promote local accumulation of Cd(II) near the electrode surface, thereby increasing the amount of metal available for electrochemical reduction and subsequent anodic stripping. This interpretation is also consistent with the hard–soft acid–base concept, since Cd(II) behaves as a relatively soft Lewis acid and exhibits favorable affinity toward sulfur-containing donor groups [21]. However, because the density of accessible thiol groups and Cd–S interactions were not directly quantified, this mechanism should be considered a chemically supported interpretation rather than direct evidence of specific molecular binding.

The amount of GO deposited on the working electrode also influenced the electrochemical response. Among the evaluated concentrations, 0.2 mg mL^−1^ produced the highest current after cysteamine functionalization, whereas both 0.1 and 0.5 mg mL^−1^ generated lower responses. At low GO loading, incomplete or heterogeneous surface coverage may limit the number of functional groups available for subsequent modification. Conversely, increasing the GO loading can generate thicker or more compact films that increase the distance for charge transport and partially restrict accessibility to the underlying conductive substrate [17,18]. This interpretation is consistent with the intermediate GO concentration providing the most favorable balance under the present experimental conditions. However, because interfacial resistance was not directly measured by electrochemical impedance spectroscopy, the decreased response at 0.5 mg mL^−1^ should not be attributed exclusively to increased charge-transfer resistance.

Raman spectroscopy provided complementary information regarding structural changes in the graphene-derived materials. The (I_D_/I_G_) ratio increased from 0.94 for GO to 1.08 for rGO and 1.15 for rGO/cysteamine. In graphene-derived carbon materials, an increase in (I_D_/I_G_) following reduction is commonly associated with changes in the size and distribution of sp^2^ domains and with the generation or exposure of additional defect and edge sites [28,29]. Accordingly, the observed increase should not be interpreted simply as deterioration of the carbon structure, but rather as evidence of structural reorganization during reduction and subsequent surface treatment. The lamellar, folded, and heterogeneous morphology observed by SEM is also characteristic of graphene oxide and provides a high-area carbonaceous interface suitable for electrochemical modification [17,22]. Complementary FTIR-ATR analysis showed spectral changes after cysteamine treatment, particularly in the C–O/C–O–C, C–N/C–O, C=C/carbonyl-related, and O–H/N–H regions. These differences between SPCE/GO and SPCE/GO–cysteamine support chemical modification of the GO-coated surface following cysteamine treatment. Nevertheless, FTIR is considered supporting evidence rather than unequivocal proof of covalent attachment; surface-sensitive techniques such as XPS would provide more direct information on nitrogen- and sulfur-containing species.

Under the selected SWASV conditions, the SPCE/GO–cysteamine platform responded to Cd(II) over the investigated range of 1–10 µg L^−1^ in standard solutions, with a sensitivity of 11.30 µA (µg L^−1^)^−1^, (R^2^ = 0.9893), an LOD of 0.25 µg L^−1^, and an LOQ of 0.74 µg L^−1^. The resulting detection limit is not the lowest reported for electrochemical Cd sensors (Table 2); however, its magnitude is compatible with portable stripping-voltammetric platforms reported in the literature. For example, Huangfu et al. reported an LOD of 0.042 µg L^−1^ using a bismuth/graphene–poly(styrenesulfonate) screen-printed electrode [30,31], while a cysteamine-modified SPCE developed by Tiwari and Kadu achieved an LOD of 0.882 nM, corresponding to approximately 0.10 µg L^−1^ Cd(II) [20]. More recently, Wang et al. obtained an LOD of 0.470 µg L^−1^ for Cd(II) after adapting an N-rGO/polypyrrole/bismuth sensing strategy to a commercial SPE [15]. In contrast, a portable bismuth/pre-anodized SPCE system reported by Li et al. showed an LOD of 3.55 µg L^−1^ when operated with a low-cost portable potentiostat [32,33]. Therefore, the analytical performance of the present platform lies within the range reported for portable electrochemical approaches, while retaining the advantage of a comparatively simple carbon/GO/cysteamine interface without an additional metallic film.

**Table 2:** Comparison of analytical performance of graphene-based electrochemical sensors for Cd(II) detection.

Sensor Material	Technique	LOD Cd(II)	Matrix	Ref.
rGO (unfunctionalized)	DPASV	0.042 µg L^−1^	Aqueous solution	[30]
rGO-Nafion	SWASV	0.10 µg L^−1^	Water	[20]
GO-Cysteine	DPV	0.470 µg L^−1^	Water	[15]
rGO-Chitosan	DPASV	0.355 µg L^−1^	Water	[33]
SPCE/GO–cysteamine	SWASV	0.25 µg L^−1^	Standard solution	This work
SPCE/GO–cysteamine	SWASV	0.56 µg L^−1^	Fortified mango digestate	This work
SPCE/GO–cysteamine	SWASV	0.17 µg L^−1^	Fortified avocado digestate	This work

Note: The LODs for the present work are reported in the measurement solution or final digestate to enable comparison in common concentration units. Sample-mass equivalents are discussed separately. The approximate conversion of 0.882 nM Cd(II) to µg L^−1^ was calculated using the molar mass of Cd.

A particularly relevant observation was the marked difference between the calibration slopes obtained in aqueous standards and in the two fruit digestates. The sensitivity decreased from 11.30 µA (µg L^−1^)^−1^ in standard solution to 5.35 µA (µg L^−1^)^−1^ in mango digestate, corresponding to a decrease of approximately 53%. In contrast, the avocado digestates produced a slope of 18.30 µA (µg L^−1^)^−1^, approximately 62% higher than the aqueous calibration and 3.4 times higher than the mango response. These results provide direct evidence that calibration behavior depends on the analytical matrix. Mango and avocado differ substantially in their original chemical composition, with mango being rich in carbohydrates, organic acids, and phenolic compounds [24,26], whereas Hass avocado has a particularly high lipid content in addition to fiber and other phytochemicals [25]. Although acid digestion removes most of the original organic material, differences in the residual composition, ionic environment, background response, and metal speciation of the final digestates may still affect Cd deposition and stripping. Matrix-dependent electrochemical behavior is well recognized as an important challenge in transferring graphene-based sensors from model solutions to food analysis [13,23].

The RSD values obtained for the fortified digestates remained below 5%, ranging from 1.50 to 3.50% for mango and from 1.60 to 4.00% for avocado. These results indicate satisfactory within-condition repeatability under the evaluated conditions. Five replicate measurements (*n* = 5) were performed at each fortification level. Moreover, because Cd(II) was added to the digestates after completion of the digestion procedure, these experiments characterize the electrochemical response in the presence of the digested fruit matrix but do not represent recovery of the entire analytical procedure. Full method validation will require fortification before digestion, analysis of independent electrodes and sample preparations, and comparison with reference techniques such as ICP-MS, ICP-OES, AAS, or GFAAS. Such validation is particularly important for food applications, where sample preparation and matrix effects can contribute substantially to total analytical uncertainty [9,13,23].

After accounting for the 0.500 g dry-sample mass and the 25.0 mL final digest volume, the estimated LOD and LOQ corresponded to 0.0133 and 0.0404 mg kg^−1^, respectively, for mango, and 0.00390 and 0.0118 mg kg^−1^ for avocado. These values refer to the dry sample mass used for digestion. This distinction is essential when considering regulatory relevance. Commission Regulation (EU) 2023/915 specifies maximum Cd concentrations on a wet-weight basis after separation of the edible portion: 0.020 mg kg^−1^ for mango and 0.050 mg kg^−1^ for fruits not included in the more restrictive categories, which includes avocado [7]. Consequently, a direct comparison between the present dry-mass analytical limits and the regulatory fresh-weight maximum levels is not valid without experimentally determined moisture contents and conversion to a fresh-weight basis. The current results should therefore be interpreted as analytical performance parameters rather than evidence of regulatory compliance. This conservative interpretation is also appropriate given the regional variability of Cd reported in Peruvian avocado fruits and production soils [5,27].

From an application perspective, the results demonstrate that the C-110/GO–cysteamine architecture can generate concentration-dependent Cd(II) responses in two digested fruit matrices with substantially different calibration characteristics. This extends the evaluation beyond aqueous standards and addresses a recognized challenge in electrochemical food analysis: preserving analytical response after transfer to complex sample matrices [13,23]. Nevertheless, further development is required before routine quantitative application. Priority studies should include matrix-matched blank measurements for LOD and LOQ estimation, pre-digestion recovery experiments at several concentration levels, selectivity tests against potentially coexisting ions such as Pb(II), Cu(II), Zn(II), and Fe(III), inter-electrode and inter-day precision, storage stability, robustness toward deposition potential, deposition time and solution pH, and direct comparison against an established spectrometric method [15,19,20]. Determination of fruit moisture content will also be required if the method is to be evaluated directly against fresh-weight regulatory limits.

## 5. Conclusions

A screen-printed carbon electrode modified with graphene oxide and cysteamine was developed for the SWASV determination of Cd(II). Comparison of the two surface-modification strategies showed that direct GO-cysteamine functionalization produced a higher cathodic response than reduction in GO prior to functionalization, while 0.2 mg mL^−1^ GO provided the highest response among the investigated loadings. The platform exhibited a concentration-dependent response between 1 and 10 µg L^−1^ Cd(II) in standard solutions, with a sensitivity of 11.30 µA (µg L^−1^)^−1^, (R^2^ = 0.9893), an LOD of 0.25 µg L^−1^, and an LOQ of 0.74 µg L^−1^. Fortified mango and avocado digestate retained concentration-dependent responses with RSD values below 5%, although the calibration slopes differed markedly between matrices, demonstrating a relevant matrix dependence. The estimated dry-sample LOD/LOQ values were 0.0278/0.0841 mg kg^−1^ for mango and 0.00866/0.0262 mg kg^−1^ for avocado. Overall, the SPCE/GO–cysteamine platform provides a promising basis for Cd(II) screening in digested fruit matrices; however, full analytical validation, including pre-digestion recovery, matrix-matched blanks, selectivity, inter-electrode reproducibility, reference-method comparison, and conversion to a fresh-weight basis, is required before its use for regulatory quantitative analysis.

## Figures and Tables

**Table 1:** Summary of analytical performance parameters for Cd(II) determination in different matrices.

Parameter	Standard Solutions	Kent Mango	Hass Avocado
Evaluated calibration interval (ppb)	1–10	1–3	1–4
Slope (µA/ppb)	11.29	5.35	18.29
R^2^	0.99	0.98	0.98
RSD (%)	—	1.50–3.5	1.60–4.00
LOD (ppb, digestate)	0.25	0.56	0.17
LOQ (ppb, digestate)	0.74	1.68	0.52
LOD (mg/kg, fruit)	—	0.03	0.009
LOQ (mg/kg, fruit)	—	0.08	0.03

## Data Availability

Data Availability Statement: The data presented in this study are available on request from the corresponding author.

## Supplementary Materials

The following supporting information can be downloaded at https://www.mdpi.com/article/10.3390/bios16100544/s1: Table S1: Comparison of Electrochemical Parameters of Functionalized Electrodes: GO—rGO—Cysteamine vs. GO—Cysteamine; Table S2: Analytical performance data for the determination of Cd+2 using SWASV on the SPE C-110 platform modified with GO/cysteamine, in the range of 1 to 10 µg·L^−1^; Table S3: Electrochemical Response and Accuracy of the C-110 Sensor, Modified with GO/Cysteamine, for the Determination of Cd+2 in Fortified Kent Mango Digestates; Table S4: Electrochemical Response and Accuracy of the C-110 Sensor, Modified with GO/Cysteamine, for the Determination of Cd+2 in Fortified Hass Avocado Digestates; Table S5: Estimated Raman intensities and ID/IG. Figure S1: FTIR spectra of bare SPCE C-110 (a), SPCE/GO (b), and SPCE/GO–cysteamine (c). The main spectral regions associated with C–O/C–O–C, C–N/C–O, C=C/carbonyl-related vibrations, and O–H/N–H are indicated.
