# Supplementary material for: GO/Cysteamine-Modified Screen-Printed Carbon Electrode for Square-Wave Anodic Stripping Voltammetric Determination of Cadmium in Fortified Mango and Avocado Digestates
Source: Biosensors (Basel). 2026 Sep 29;16(10):544. doi: 10.3390/bios16100544 (PMC13702389; doi:10.3390/bios16100544)

Supplementary material

GO/Cysteamine-Modified Screen-Printed Carbon Electrode for Square-Wave Anodic Stripping Voltammetric Determination of Cadmium in Fortified Mango and Avocado Digestates

Table S1. Comparison of Electrochemical Parameters of Functionalized Electrodes: GO - rGO - Cysteamine vs. GO – Cysteamine.

**Observed voltammetric parameter**	**GO - rGO – Cys**	**GO - Cys**
Anodic Peak Current (I_{pa}) (μA)	+80 to +90	+80 to +90
Cathodic Peak Current (I_{pc}) (μA)	-30 to -50	-70 to -90
Anodic Peak Potential (E_{pa}) (V)	+0.2 to 0.3	+0.05 to +0.20
Cathodic Peak Potential (E_{pc}) (V)	≈ -0.05	-0.05 to -0.10
Apparent reversibility	Quasi-irreversible	Quasi-irreversible

Table S2. Analytical performance data for the determination of Cd^{+2} using SWASV on the SPE C-110 platform modified with GO/cysteamine, in the range of 1 to 10 μg·L⁻¹.

Rep.	Blank	Current intensities for different cadmium concentrations (ppb)									
	HCl - 0.05M	1ppb	2ppb	3ppb	4ppb	5ppb	6ppb	7ppb	8ppb	9ppb	10ppb
1	16.244	38.572	48.409	58.324	68.317	78.386	88.534	98.759	109.062	119.442	140.124
2	17.270	39.736	49.767	59.838	69.946	80.094	90.28	100.505	110.769	121.071	141.638
3	17.737	40.9	51.125	61.351	71.576	81.801	92.026	102.251	112.476	122.701	143.151
4	18.110	42.064	52.483	62.864	73.206	83.508	93.772	103.997	114.183	124.331	144.664
5	18.390	43.228	53.841	64.378	74.835	85.216	95.518	105.743	115.89	125.96	146.178
Prom.	17.55	40.90	51.13	61.35	71.58	81.80	92.03	102.25	112.48	122.70	143.15
SD	0.84										

Table S3. Electrochemical Response of the C-110 Sensor, Modified with GO/Cysteamine, for the Determination of Cd^{+2} in Fortified Kent Mango Digestates.

Rep.	Blank	Current intensities for different cadmium concentrations (ppb) for Kent Mango		
	HCl - 0.05M	1ppb	2ppb	3ppb
1	19.67	26.5	32.1	35.9
2	20.28	27.7	32.6	36.2
3	18.75	25.9	31.23	35.3
4	19.98	27.1	32.35	36.05
5	21.22	28.3	33.47	36.8
Prom.	19.98	27.10	32.35	36.05
SD	0.90	0.95	0.81	0.54
%RSD	4.50	3.50	2.51	1.50

Table S4. Electrochemical Response of the C-110 Sensor, Modified with GO/Cysteamine, for the Determination of Cd^{+2} in Fortified Hass Avocado Digestates.

Rep.	Blank	Current intensities for different cadmium concentrations (ppb) of Hass avocado.			
	HCl - 0.05M	1ppb	2ppb	3ppb	4ppb
1	19.67	51.1	62.2	83.6	96.6
2	20.28	47.8	60.3	79.1	94.4
3	18.66	47.19	58.65	79.75	93.64
4	19.98	49.45	61.25	81.35	95.5
5	21.30	51.71	63.85	82.95	97.36
Prom.	19.97	49.45	61.25	81.35	95.50
SD	0.96	1.98	1.96	1.95	1.53
%RSD	4.79	4.00	3.19	2.40	1.60

Table S5. Estimated Raman intensities and I_D/I_G.

**Sample**	**I_D (A.U)**	**I_G (A.U)**	**I_D/I_G**
GO	1280	1360	0.94
rGO	860	800	1.08
rGO/cysteamine	830	720	1.15

Figure S1. FTIR spectra of bare SPCE C-110 (a), SPCE/GO (b), and SPCE/GO–cysteamine (c). The main spectral regions associated with C–O/C–O–C, C–N/C–O, C=C/carbonyl-related vibrations, and O–H/N–H are indicated.

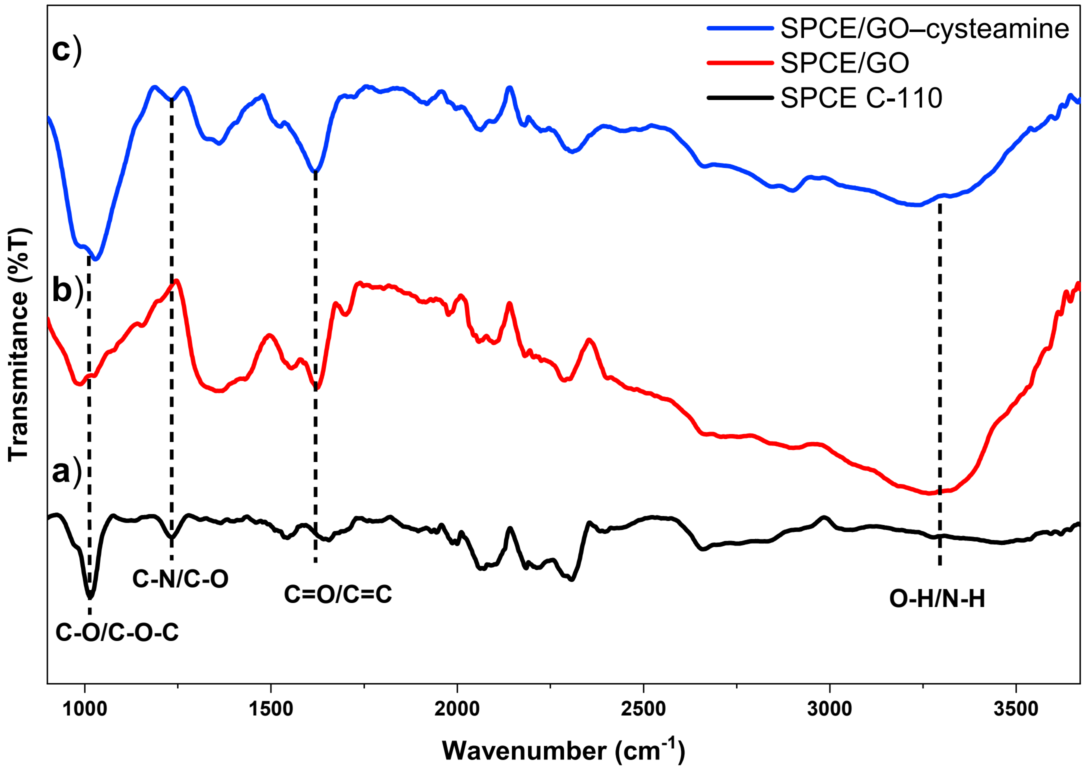

Supplement: Supplementary file 1 [file biosensors-16-00544-s001.zip › biosensors-4555460-supplementary.pdf]
